# Age-dependent changes in intuitive and deliberative cooperation

**DOI:** 10.1038/s41598-023-31691-9

**Published:** 2023-03-17

**Authors:** Francesco Nava, Francesco Margoni, Nilmini Herath, Elena Nava

**Affiliations:** 1 Department of Economics, London School of Economics, London, UK; 2 Department of Psychology, University of Oslo, Oslo, Norway; 3 Department of Social Studies, University of Stavanger, Stavanger, Norway; 4 Department of Psychology, University of Milano-Bicocca, Piazza Dell’Ateneo Nuovo 1, 20126 Milan, Italy

**Keywords:** Psychology, Human behaviour

## Abstract

Cooperation is one of the most advantageous strategies to have evolved in small- and large-scale human societies, often considered essential to their success or survival. We investigated how cooperation and the mechanisms influencing it change across the lifespan, by assessing cooperative choices from adolescence to old age (12–79 years, N = 382) forcing participants to decide either intuitively or deliberatively through the use of randomised time constraints. As determinants of these choices, we considered participants’ level of altruism, their reciprocity expectations, their optimism, their desire to be socially accepted, and their attitude toward risk. We found that intuitive decision-making favours cooperation, but only from age 20 when a shift occurs: whereas in young adults, intuition favours cooperation, in adolescents it is reflection that favours cooperation. Participants’ decisions were shown to be rooted in their expectations about other people’s cooperative behaviour and influenced by individuals’ level of optimism about their own future, revealing that the journey to the cooperative humans we become is shaped by reciprocity expectations and individual predispositions.

## Introduction

Cooperative behaviour is central to most human enterprises, and it is a cornerstone of human societies^1,2^. Studies suggest that a rudimental and intuitive moral sense is present already in the first year of life^3–6^ and a spontaneous proclivity toward altruism, fairness and cooperation can be detected already in toddlerhood, although limited or constrained by several biases such as ingroup and familiarity preferences that might hinder impartiality to some extent^7,8^. In adults, prosociality as a default intuitive response rather than a self-reflected process is in line with findings from experimental economic games that have been used to measure cooperation, overall suggesting that humans are intuitively cooperative rather than intuitively selfish^9,10^. In one-shot public goods games, forcing participants (in particular young adults between 20 and 30 years of age) to decide quickly has been shown to increase rather than decrease cooperation^11–14^.

A possibility put forward to explain these latter findings is that humans are either equipped with or gradually develop fast and effortless cooperative heuristics, which are rational and shaped by previous experience, for instance learning that cooperating can often be payoff-maximizing in the long run^15–19^. In one-shot games though, to cooperate is not the payoff-maximizing strategy, since participants will not encounter their partners again^20^. In such scenarios, a more controlled and slow response often leads participants to rationally act more selfishly^21^. Several key elements of this proposed theory remain unclear and untested including the possibility that cooperative heuristics may change over the lifespan and may thus be shaped by experience which is then internalized through expectations about how others will likely respond to cooperation or defection.

Our analysis explores whether and how cooperative heuristics change throughout the lifespan, from early adolescence to old age, as assessed in a one-shot public goods game (PGG) played under time pressure or time delay. If it is true that cooperation results from the overgeneralization of intuitive heuristics that are internalised from experience and applied to novel contexts, one might expect to see changes in cooperative behaviour throughout the lifespan, as individuals gain more social experience with age, or simply hold different expectations about others’ relevant behaviour depending on their stage of development (e.g., adolescence vs old age).

Moreover, to test if social heuristics fostering prosocial behaviour apply to altruism too, participants were asked to play a one-shot dictator game (DG), again under time pressure or time delay. This allowed us to test whether and to what extent cooperative and altruistic behaviour are differently affected by intuition and deliberation.

Next, we investigated the role of social expectations and reciprocity in shaping cooperative behaviour, by asking participants to report how much they thought the other two (fictitious) partners in the PGG had contributed, and in a subsequent task, by manipulating the knowledge participants had about the partners’ contributions. In particular, the tight relationship between cooperation and reciprocity (both direct and indirect) has been theorized by evolutionary models and demonstrated by experimental evidence^13,22–28^.

Finally, the role of attitudes to risk, general optimism, and the need to be socially accepted were also analysed as potential sources of cooperative behaviour.

## Results

### Becoming intuitive cooperators

In Public Goods Games (PGGs), both Nash and dominant strategy equilibrium behaviour require players to free ride to maximise their short-term monetary payoff. However, cognitive accounts have shown that people’s decisions are influenced by social heuristics—intuitive judgments that are less cognitively costly than deliberate reasoning^14,29^. Whereas deliberation is flexible in nature, intuition is not and seems to support cooperation rather than selfishness in adulthood^15^. If cooperation is an evolved strategy built in humans as an intuitive response, then one may expect it to be stably present across the lifespan, at least after childhood^30,31^. To test the stability across the lifespan of the intuition for cooperation, participants were asked to play a one-shot PGG, under two different time conditions. In the time-pressure condition, participants had only 10 s to respond, whereas in the time-delay condition, participants were forced to deliberate on their choice for at least 10 s, after which they had 20 s to respond. These two conditions were selected, as in prior work^14,32^ to provide insight into the intuitive and reflective (deliberative) processes underlying participants’ choices. Forcing participants to respond quickly should elicit intuitive responses, whereas forcing them to respond slowly should elicit reflective reasoning. In the PGG, each participant was matched with two other fictitious partners, received four lottery tickets (granting the possibility to win a monetary prize), and had to decide how many tickets to contribute to a common fund. The total amount of tickets placed in the common fund was then to be doubled and divided evenly among the three players. Raw data, videos and supplementary material can be found on the Open Science Framework^33^.

**Figure 1:**
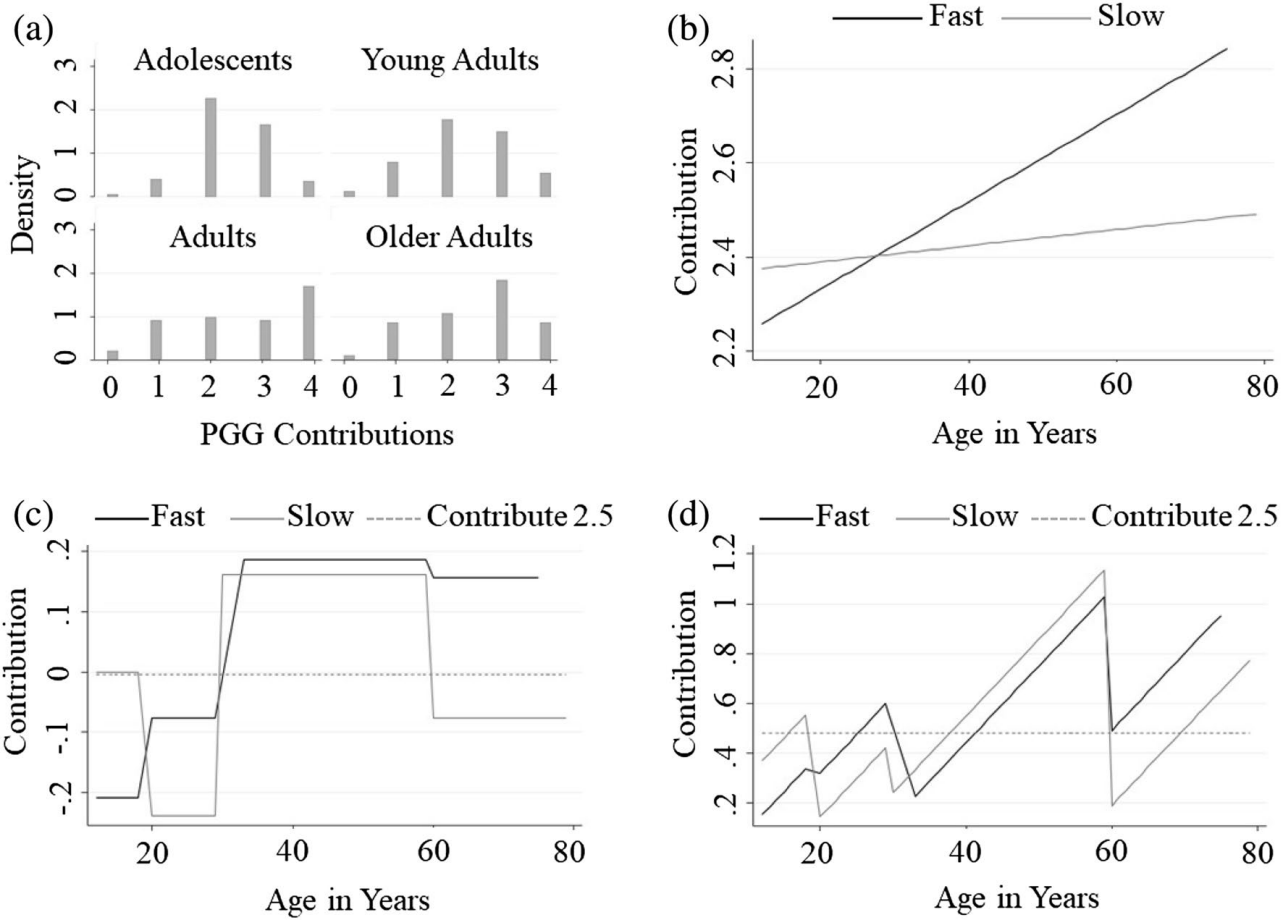
Participants’ Choices in the Public Goods Game**.** Panel (**a**) reports the frequency of contribution within the four age groups across conditions. Participant contributions increase across the lifetime only to taper off in older adults. Panel (**b**) depicts the OLS estimation of specification (1) with the black line representing estimated contributions under time-pressure (fast), and the grey line representing estimated contributions under time-delay (slow). Panels (**c**) and (**d**) depict the ordered Probit regressions by age group from Eqs. (2) and (3) with black lines depicting expected contribution under time-pressure and grey lines depicting contributions under time-delay. In both panels, the dependent variable is rescaled by the ordered Probit approach, and the dashed line represents a contribution of 2.5 lottery tickets.

Our main empirical model specification exploits a conventional ordered Probit approach. Note that our ordered Probit regressions estimate the likelihood of contributing any number of tickets via maximum likelihood, positing that PGG contributions are a continuous unobserved random variable, as a function of the regressors and of normally distributed noise, which is then parsed into the corresponding ordered discrete variable (called PGG). When estimating such regressions, thresholds for the continuous omitted variable are also estimated to associate any value for the continuous omitted variable to a specific value for the ordered discrete dependent variable. For instance, one such threshold is the line labelled “Contribute 2.5 Tickets” in Panel c of Fig. 1 which can be interpreted as stating that values above 0 for the omitted variable should be treated as contributions at least as high as 3 whereas values below 0 should be interpreted as contributions no higher than 2. We estimated the likelihood of contributing any number $N\in \{\mathrm{0,1},\dots ,4\}$ of lottery tickets as a function of age, time condition (time-pressure vs time-delay), and the interaction between time condition and age. The coefficient on the second regressor (parameter *b* in specification (1)) estimates differences in intercepts between the time-pressure and the time-delay group, whereas the coefficient on the third regressor (parameter *c* in specification (1)) estimates differences in slopes across the two groups (as displayed by the OLS regression lines in Panel b of Fig. 1). Because the regressions lines for the time-pressure and time-delay groups were close on parts of the support due to their intersection, in the interaction term of our main specification, age was divided into four categories: adolescents (12–19 years), young adults (20–30 years), adults (31–60 years), and older adults (61–79 years). This also allowed us to investigate impacts of stages of development rather than age as, although these concepts are closely related, our research question centres on the former. Specifically, we run the following ordered Probit regressions:

1 $$PGG=k+a*Age+b*Time+c*Time*Age$$

2 $$PGG=k+b*Time+c*Time*AgeGroup$$

3 $$PGG=k+a*Age+b*Time+c*Time*AgeGroup$$

where $PGG$ denotes the contribution to the public goods game, $Time$ is a binary variable taking value 1 for those under time-pressure and 0 for those in the time-delay condition, $AgeGroup$ is a categorical variable identifying the age group to which the individuals belong, and $k,a,b,c$ are the parameters to be estimated (note that in Ordered Probit regressions, the omitted dependent variable is estimated so that it is worth 0 when all regressors are worth 0, meaning that constant terms k are normalized to 0 in all Probit regressions).

The main finding of the study, as captured in Fig. 1 and Table 1, is that attitudes towards cooperation change significantly across the stages of development. The estimate of specification (1) suggests in fact that individuals under time pressure cooperate more with age, while slowly deliberated decisions are not significantly affected by age (Fig. 1b).

**Table 1:** Ordered probit estimates for specifications (1), (2) and (3).

Variables	Specification (1)	Specification (2)	Specification (3)
Time	− 0.22	0.15	− 1.35*
Age	0.002	–	0.03**
Time × age	0.008^+^	–	–
Slow × adolescent	–	(Baseline)	(Baseline)
Slow × early adult	–	− 0.24	− 0.47*
Slow × adult	–	0.16	− 0.68^+^
Slow × older adult	–	− 0.08	− 1.65**
Fast × adolescent	–	− 0.37^+^	1.13*
Fast × early adult	–	− 0.23	1.06*
Fast × adult	–	0.03	0.56^+^
Fast × older adult	–	(Omitted)	(Omitted)
Contribute 0.5	− 1.87***	− 2.01***	− 1.56***
Contribute 1.5	− 0.83***	− 0.97***	− 0.49*
Contribute 2.5	0.13	− 0.01	0.48*
Contribute 3.5	1.09***	0.95***	1.44***
Participants	(369)	(369)	(369)

The ordered Probit approach transforms the discrete dependent variable in a continuous one, and the rows in the table labelled Contribute X represent different cut-offs for the transformed dependent variable above which participants contribute more than X tickets and below which they contribute less than X tickets. Our key specification (3), displays a significant coefficient for almost all parameters and underlies the graphical representation provided in Fig. 1d (Please note that the variable “fast × older adult” is omitted from regressions because of collinearity. The coefficient on variable “slow × adolescents” is omitted from regressions because of collinearity). Qualitatively similar results hold when age is dropped as an explanatory variable, in specification (2). However, many coefficients lose significance, as interaction terms are less precisely estimated when accounting also for the age variation in PGG contributions within age groups. When age is treated as a continuous variable in the interaction term, contributions of those under time-delay do not vary with age whereas contributions of those under time pressure increase with age (although p-value on the coefficients being different is only 0.14).

^+^*p* < 0.15 (marginally significant), *p < 0.05, **p < 0.01, ***p < 0.001.

Although age had an overall positive effect on contributions for all participants in the time-pressure condition, intuitive cooperation, as predicted by prior work (that is, more cooperation under time-pressure than under time-delay; see^14^), was not present in all age groups in specifications (2) and (3). It was absent in adolescents and adults, but present in young adults and in the older adults (Fig. 1c,d). Interestingly, a reversed pattern was found in adolescents, in that they behaved more selfishly under time-pressure than when given time to reflect. Notably, whereas when aggregating across age groups we replicated prior findings^14^ this was not the case for adolescents, for whom intuition favoured selfishness, or for adults, whose contributions were unaffected by time condition. Further, as depicted in Fig. 1a and d, adults were the age group contributing the most to the public good under both time conditions, suggesting that with maturity, individuals internalize the benefits of cooperation in both intuitive and reflective processes. Finding that adolescents and adults do not display intuition for cooperation is most striking considering recent studies arguing that public goods games with low contribution equilibria, such as ours, might naturally favour the emergence of intuition for cooperation because of mistakes arising in fast decision making^34^.

When, for the sake of robustness, gender was added as an explanatory variable in specification (3), female participants were found to contribute slightly more than males (see Table 2). When predicted response times were added to the specification to check whether these influenced PGG contributions within each time condition, participants taking longer to respond were shown to contribute less to the PGG (see Table 2 and the related discussion in the Extended Data section).

**Table 2:** Ordered probit estimates of specifications (13), (14), and (15).

Variables	Specification (13)	Specification (14)	Specification (15)
Age	0.03**	0.03*	–
Female	0.22*	–	–
Certainty equivalent	–	0.06*	–
Need to belong	–	0.17^+^	–
Predicted response time	–	–	− 2.36**
Time	− 1.30*	− 1.00^+^	− 28.2**
Slow × adolescent	(Baseline)	(Baseline)	(Baseline)
Slow × early adult	− 0.47*	− 0.46*	− 0.47*
Slow × adult	− 0.66^+^	− 0.46	− 0.68^+^
Slow × older adult	1.63**	− 1.36*	− 1.65**
Fast × adolescent	1.08^+^	0.76	1.14*
Fast × early adult	1.01*	0.81^+^	1.06*
Fast × adult	0.54^+^	0.34	0.57^+^
Fast × older adult	(Omitted)	(Omitted)	(Omitted)
Contribute 0.5	− 1.41***	− 0.83^+^	− 41.3***
Contribute 1.5	− 0.33	0.25	− 40.3***
Contribute 2.5	0.65*	1.23**	− 39.3***
Contribute 3.5	1.60***	2.20***	− 38.3***
Participants	(367)	(339)	(369)

^+^p < 0.15 (marginally significant), *p < 0.05, **p < 0.01, ***p < 0.001.

### The coincidence of cooperative behaviour and expected reciprocity

Reciprocity provides a powerful mechanism for promoting and sustaining cooperation, even when expectations might prove to be overly optimistic^35–38^. Further, the manipulation of participants’ beliefs about the partner’s intention to cooperate has been shown to directly modulate participants’ behaviour: for instance, they were shown to speed up cooperation and slow down defection^39^. To explore the role of reciprocity across the lifespan, we began by eliciting beliefs about the contributions of others by asking participants to estimate the contribution of their two partners after playing the PGG. These two estimates were then averaged to construct a variable, called *expected cooperation*, aimed at capturing the participant’s expectations about the cooperativeness of others.

Expected cooperation was found to be so closely associated to PGG contributions that running a simple OLS regression with PGG contributions as the dependent variable and expected cooperation as the only explanatory variable resulted in an *R*^*2*^ coefficient of 0.86. Interestingly, participants contributed on average 6% more than what they expected others to contribute (with the coefficient on the expected cooperation variable strictly exceeded unity with 99% significance). Expected cooperation was then added to our baseline specification (3) as an explanatory variable to test whether age and time shape PGG contributions beyond determining the level of cooperation expected from others, resulting in the following ordered Probit regression:

4 $$PGG=k+a*Age+b*Time+c*Time*AgeGroup+d*ExpectedCooperation$$

Findings reported in column (4) of Table 3 establish that in such a specification, expected cooperation remained the only significant variable in explaining PGG contributions. In essence, age and time were shown to co-determine expectations about the cooperation of others as well as PGG contributions. One may speculate that time and age first determine beliefs about PGG contributions of partners which in turn determine participants’ PGG contributions. To further highlight the fact that age and time jointly determine PGG contributions as well as expected cooperation, in specification (5) of Table 3, we ran the same regression as in specification (3), but with expected cooperation as a dependent variable instead of PGG contributions:

**Table 3:** Ordered probit estimates of specifications (4), (5) and (6).

Variables	Specification (4)	Specification (5)	Specification (6)
Time	− 0.63	− 1.50**	− 1.36*
Age	0.02	0.03**	0.03*
Expected cooperation	0.74***	–	–
Optimism	–	–	− 0.01*
Slow × adolescent	(Baseline)	(Baseline)	(Baseline)
Slow × early adult	− 0.24	− 0.38^+^	− 0.51^+^
Slow × adult	− 0.15	− 0.96**	− 0.69*
Slow × older adult	− 0.88	− 1.80**	− 1.63**
Fast × adolescent	0.57	1.22*	1.11*
Fast × early adult	0.58	1.11*	1.09*
Fast × adult	0.51	0.29	0.59*
Fast × older adult	(Omitted)	(Omitted)	(Omitted)
Contribute 0.5	− 0.42	− 1.53***	− 2.06***
Contribute 1.5	− 0.76**	− 0.73**	− 1.01**
Contribute 2.5	1.89***	0.71**	− 0.03
Contribute 3.5	3.05***	1.66***	0.93**
Participants	(369)	(369)	(357)

^+^p < 0.15 (marginally significant), *p < 0.05, **p < 0.01, ***p < 0.001.

5 $$Expected Cooperation=k+a*Age+b*Time+c*Time*AgeGroup$$

As evident from Table 3, coefficients in specification (5) were minimally affected relative to those reported specification (3) of Table 1.

### Optimism as a determinant of cooperative behaviour and beliefs about cooperation

A measure of general optimism was then elicited from participants to test if it increases expected cooperation and in turn PGG contributions. Participants were asked to estimate the likelihood that certain negative events might happen to them in the future (knowing the proportion of the general population experiencing such negative outcomes). It is well documented that individuals tend to underestimate the subjective likelihood of such negative events, even when the actual probabilities are low, a phenomenon known as *optimism bias*^40^.

An index of optimism was constructed by averaging participants’ estimates of their subjective probability of experiencing negative events in the future (ranging in the data from 0 to 60 with lower values for the index revealing a more optimistic outlook). Two simple OLS regression established that optimism was associated with expected cooperation and PGG contributions, with more optimistic individuals expecting others to contribute more (see Table 4). Further analyses revealed age being a positive determinant of optimism (see Table 5), and optimism being a positive determinant of PGG contributions even holding fixed age and time, see column (6) of Table 3 where the following Probit specification was estimated:

**Table 4:** OLS estimates with PGG contributions and expected cooperation as dependent variables and optimism and a constant as regressors.

Variables	Expected cooperation	PGG
Constant	2.55***	2.90***
Optimism	− 0.010**	− 0.011**

**p < 0.01, ***p < 0.001.

**Table 5:** OLS estimates with optimism as dependent variable and age and a constant as regressors.

Variables	Optimism
Constant	46.0***
Age	− 0.18***

***p < 0.001.

6 $$PGG=k+a*Age+b*Time+c*Time*AgeGroup+d*Optimism$$

### Conditional cooperation as an intuitive process

To verify the extent to which individuals explicitly reciprocate in the PPG, participants were asked to play the same PGG but this time aware of how much their two other partners would usually contribute (and therefore, will likely have contributed). Contrary to the manipulation conducted when eliciting cooperative expectations about partners, these tasks were designed to explicitly assess the intention to cooperate given prior information about other partners’ actual behaviour.

Participants were presented with five informational settings, ranging from low contributions from both partners to high contributions from both partners, and including unequal contributions from the two partners. We analysed the effects of such informational manipulations on PGG contributions to understand to which extent conditional cooperation is an intuitive rather than deliberative process and how it is impacted by age. OLS regressions were run to estimate the following three models for conditional cooperation:

7 $$Resp\_xy=c*PGG$$

8 $$Resp\_xy=a*Time+c*PGG$$

9 $$Resp\_xy=k+b*Age+c*PGG$$

where $Resp\_xy$ denotes the PGG contribution of a participant who knows that their two partners likely contributed respectively $x$ and $y$ tickets, and $PGG$ denotes the contribution when the participant does not know the contribution of their partners.

When partners’ contributions were low (i.e., contributions of 0 and 1), participants contributed only 61% of what they had contributed to the original PGG. In the three settings in which the average partners’ contributions were equal to 2 (i.e., contributions of either 2 and 2, or 3 and 1, or 0 and 4), participants contributed 81% of what they had contributed to the original PGG. Finally, when partners’ contributions were high (i.e., contributions of 3 and 4), participants contributed as in the original PGG. These associations were estimated by running OLS regressions without constants, having the original contribution (standard PGG) as the only independent variable and the new contribution (conditional) as the dependent one (see Table 6 for further details). These results showcase both reciprocity as a key determinant of contributions and the intrinsic optimism of participants about the behaviour of their partners in the original uninformed PGG.

**Table 6:** OLS Estimates for Specification (7).

Variables	Resp_0–1	Resp_2–2	Resp_3–4	Resp_0–4	Resp_3–1
PGG	0.61***	0.81***	1.00***	0.81***	0.81***

Resp_0–1 describes contributions after being told that partner A contributed 0 tickets, and that B contributed 1 ticket. Resp_2–2 describes contributions after being told that both partners contributed 2 tickets. Resp_3–4 describes contributions after being told that partner A contributed 3 tickets and that B contributed 4 tickets. Resp_0–4 describes contributions after being told that partner A contributed 0 tickets and that B contributed 4 tickets. Resp_3–1 describes contributions after being told that partner A contributed 3 tickets and that B contributed 1 ticket.

***p < 0.001.

When time was added as an explanatory variable in specification (8), time-pressure was found to have a positive effect on cooperation. Irrespective of the contributions given by partners, intuition favoured cooperation. Specifically, participants in the time-delay condition contributed less in the informed settings than in the uniformed one, even when faced with the most generous partners who were contributing 3.5 tickets on average (see Table 7). Instead, relative to the original uniformed PGG, those in the time-pressure condition contributed more when faced with the most generous partners (who contributed on average 3.5 tickets), less when faced with the most selfish partners (who were contributing on average 0.5) and comparably when faced with partners donating on average 2 tickets.

**Table 7:** OLS estimates for specification (8).

Variables	Resp_0–1	Resp_2–2	Resp_3–4	Resp_0–4	Resp_3–1
Time	0.21*	0.34***	0.43***	0.31**	0.39***
PGG	0.57***	0.75***	0.92***	0.76***	0.74***

*p < 0.05, **p < 0.01, ***p < 0.001.

By further adding a constant and age as explanatory variables in specification (9), the key insights remained largely unaffected. However, age was found to be positively associated with the contributions across the five informed settings (and significantly so in two of them) even accounting for the fact that contributions in the original PGG were already increasing with age (see Table 8). Adding constants had a profound effect on the estimation by differentiating the three settings in which the average partners’ contributions amounted to 2. More equitable contributions by partners (2–2 and 3–1) were found to have a higher constant and a less pronounced dependence on the original PGG contribution than the setting in which contributions were most inequitable (0–4), revealing that inequality in partners’ contributions led to more sensitive responses by participants (see Tables 8).

**Table 8:** OLS estimates for specifications (9).

Variables	Resp_0–1	Resp_2–2	Resp_3–4	Resp_0–4	Resp_3–1
Constant	0.31^+^	1.04***	1.45***	0.62***	0.96***
PGG	0.46***	0.42***	0.47***	0.51***	0.40***
Age	0.004	0.002	0.001	0.007*	0.006*

^+^p < 0.15 (marginally significant), *p < 0.05, **p < 0.01, ***p < 0.001.

### The rise of altruism from adulthood to old age

To test if pure generosity, or altruism, contributes to intuitive cooperative behaviour, participants were asked to play a dictator game in which they chose how much to donate to others, knowing that they would not receive anything in return (under the same time conditions used in the PGG). Dictator games have been used traditionally to measure pure altruism rather than cooperative behaviour^41^. Behaviourally stark differences appear across these two settings despite incentives coinciding and selfish behaviour being strategically optimal in both tasks.

As in PGG, the main empirical model specifications consisted of ordered Probit regressions in which the likelihood of donating any number $N\in \{\mathrm{0,1},\dots ,4\}$ of lottery tickets was explained by age, time condition, and the interaction between the two. The following ordered Probit regressions were run (as in the original PGG):

10 $$DG=k+a*Age+b*Time+c*Time*Age$$

11 $$DG=k+b*Time+c*Time*AgeGroup$$

12 $$DG=k+a*Age+b*Time+c*Time*AgeGroup$$

where $DG$ denotes the donation of the participant in the dictator game.

**Figure 2:**
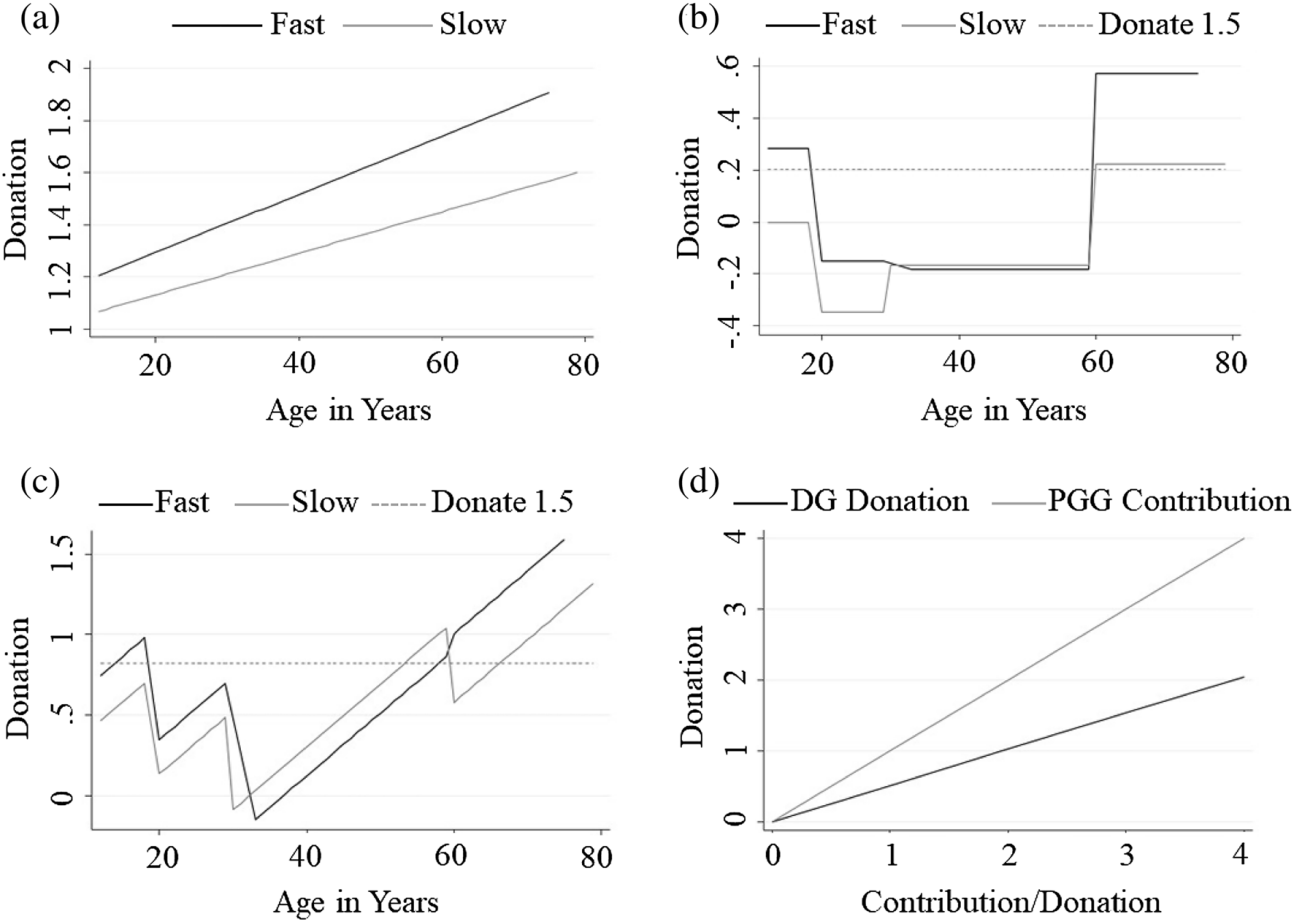
Participants’ Choices in the Dictator Game. Panel (**a**) depicts the OLS estimation of specification (10), with the black line representing estimated donations under time-pressure (fast), and grey line representing estimated donations in the time-delay (slow). Panels (**b**) and (**c**) depict the ordered Probit regressions by age group from Eqs. (11) and (12), with black lines depicting expected donations under time-pressure and grey lines depicting donations under time-delay. In both panels, the dependent variable is rescaled by the ordered Probit approach, and the dashed line represents a donation of 1.5 lottery tickets. The black line in Panel (**d**) depicts estimated DG donations as a function of PGG contribution, showing that on average individuals donated in the DG 51% of what they contributed in the PGG.

As in the PGG, age had a positive and significant effect on donations. In the baseline specification (10), individuals were shown to donate more as they grew older with donations increasing over the lifespan by as much as 60% (see Fig. 2a). Probit specifications (11) and (12) by age group further revealed that altruism first declines from adolescence until adult age, and then increases rapidly and consistently across adulthood and old age, as can be seen in Fig. 2b and c (for details see Table 9). The pattern is notably different from behaviour in the PGG in that PGG contributions increased till adulthood only to decline in old age, whereas DG donations declined till adulthood only to increase thereafter. Further, time-pressure had a positive impact on DG donations across age groups (except for adults), whereas PGG contribution were diminished by time-pressure in adolescence. This suggests that altruism as an intuitive behavioural response present from adolescence. A caveat to this observation is the behaviour of adults where the time condition had a very limited impact on DG donations. This was also the case in PGG, where contributions of adults were only marginally affected by the time condition. One may conjecture that in both games, adults followed well-established norms of behaviour irrespective of the time given for a decision, entailing high levels of cooperation in public goods setting, and low levels of altruism in dictator setting.

**Table 9:** Ordered probit estimates for specifications (10), (11), and (12).

Variables	Specification (10)	Specification (11)	Specification (12)
Time	0.17	0.57**	− 1.32*
Age	0.01*	–	0.04***
Time condition × age	0.00	–	–
Slow × adolescent	–	(baseline)	(baseline)
Slow × early adult	–	− 0.35^+^	− 0.64**
Slow × adult	–	− 0.17	− 1.25**
Slow × older adult	–	0.22	− 1.75**
Fast × adolescent	–	− 0.29*	1.61**
Fast × early adult	–	− 0.73***	0.90^+^
Fast × adult	–	− 0.76**	− 0.10
Fast × older adult	–	(omitted)	(omitted)
Donate 0.5	− 0.23	− 0.63***	− 0.03
Donate 1.5	0.59***	0.21	0.81***
Donate 2.5	1.49***	1.13***	1.76***
Donate 3.5	2.00***	1.66***	2.28***
Participants	(370)	(370)	(370)

^+^p < 0.15 (marginally significant), *p < 0.05, **p < 0.01, ***p < 0.001.

Finally, we ran a simple OLS regression with DG donations as a dependent variable and PGG contributions as the only explanatory variable (see Fig. 2d). Participants in the DG donated on average 51% of what they were contributing in the PGG with an *R*^*2*^ = 0.59. Such a stark difference in behaviour across the two tasks suggests that participants clearly understood the difference between them (the potential benefits of cooperating in the PGG vs the act of donating without receiving anything in return in the DG).

Two additional specifications then established that DG donations significantly increased PGG contributions above and beyond the effects of age, time, and their interaction (see Table 10 for details).

**Table 10:** Ordered probit estimates of specifications (16) and (17).

Variables	Specification (16)	Specification (17)
Time condition	− 1.10^+^	− 0.79
Age	0.02*	0.02^+^
DG	0.27***	0.26***
Need to belong	–	0.04
Certainty equivalent	–	0.19^+^
Slow × adolescent	(baseline)	(baseline)
Slow × early adult	− 0.32^+^	− 0.30
Slow × adult	− 0.40	− 0.22
Slow × older adult	− 1.27*	− 1.04^+^
Fast × adolescent	0.77	0.47
Fast × early adult	0.90^+^	0.69
Fast × adult	0.67*	0.46^+^
Fast × older adult	(omitted)	(omitted)
Contribute 0.5	− 1.42***	− 0.71
Contribute 1.5	− 0.30	0.42
Contribute 2.5	0.71**	1.44**
Contribute 3.5	1.70***	2.44***
Participants	(364)	(339)

^+^p < 0.15 (marginally significant), *p < 0.05, **p < 0.01, ***p < 0.001.

### Risk aversion and desire for social acceptance as determinants of cooperation

Measures of risk aversion and of desire for social acceptance (i.e., need to belong) were added to the main specification (3). Adding elicited certainty equivalents (a measure of risk aversion that we captured using a standard task, see^42^) and need-to-belong responses (a measure of desire for acceptance that we captured using a standard survey, see^43^) as regressors to our baseline established that more risk averse individuals (i.e. those with a lower certainty equivalent) contributed less to the public good than individuals with lower risk aversion (both conditioning on DG donations and not) suggesting that participants perceived contributing as a risky option (see Tables 2 and 10). Participants caring more about social acceptance contributed more to the public good (see Table 2).

## Discussion

According to recent dual-process accounts of cooperative decision making, deliberative self-control sustains selfish impulses to maximise an individual’s payoff, whereas cooperative and altruistic behaviour is favoured by intuition and social heuristics^9,10,14^; for a review, see^16^. The current results reveal important changes in cooperative behaviour throughout the lifespan, overall suggesting that an intuition for cooperation may not be the default in all stages of life. We found that the strength of an intuitive response favouring cooperation increases with age but outperforms self-reflected behaviour only with adulthood (Fig. 1b). Indeed, adolescents contribute more in the PGG when asked to reason about their choice than when they were forced to respond quickly, a result that is in stark contrast with the hypothesis that intuition supports cooperation in this age group (but see^44^). Adolescents seem to be intuitively selfish.

Intriguingly, inspection of Fig. 1c reveals that intuitive and deliberative processes underlying cooperation do not change linearly across the lifespan. It appears in fact that from youth until adulthood the intuitive processes lead to norms that are ever more cooperative as individuals most likely learn by experience about the benefits of cooperative behaviour. Instead, deliberative processes appear to follow a quite distinct developmental pattern—with reflection favouring cooperation in adolescence relative to the instinctive norm and hindering cooperation in early adulthood and old age. Adults instead appear so ingrained in their cooperative and altruistic norms of behaviour that decision time simply does not affect their choices, with such norms being most cooperative and least altruistic among all age groups (see Figs. 1a and 2b). These patterns suggest the possibility that it is through experience and exposure to cooperative environments over the lifespan that individuals learn about the benefits of cooperating and instinctively contribute more than through deliberation. While our findings would be consistent with such a possibility, the role that experience plays in shaping the development of cooperative behaviour remains to be investigated.

Our second main result establishes that the cooperative behaviour of individuals is largely driven by the expectations or beliefs about the cooperative behaviour of partners. This does not change throughout the lifespan: adolescents, adults and elderly people consistently contribute 6% more than what they expect others to contribute, irrespective of time constraints. In essence, age and decision time appear to co-determine both the level of cooperation expected from partners and the contributions in the PGG. One could speculate that in the construction of a hierarchy of processes, age and intuition might first modulate the beliefs about other people’s cooperative behaviour, eventually determining an individual’s cooperative choices (a proposal that would be consistent with prior work, see^13,35,45^).

The fact that all participants contributed essentially what they believed others would contribute suggests that two aspects further characterize cooperation: first, that throughout the lifespan, humans expect to be reciprocated, which is in line with developmental studies revealing that the norm of reciprocity is acquired already during the preschool years^45–47^, with findings even suggesting that an initial core understanding of reciprocity might be present in infancy^48,49^; second, that throughout the lifespan, beliefs about other people’s decisions are modulated by intuition and deliberation in the same way that their own decisions are. Overall, our results are in line with a more general reciprocity account of cooperation, by which both direct and indirect reciprocity (more than other strategies such as conformity^50^) might have promoted the evolution of cooperation^1,28,51^.

We also found that optimism shapes expectations about partners’ contributions, so that more optimistic individuals acted more cooperatively in the PGG themselves and also expected their partners to be more cooperative^52^. Importantly, our data show that optimism grows with age, which can partly account for the reflective approach to cooperation in adolescence. A possibility here is that younger individuals are less optimistic about their future and about the behaviour of others, which in turn pushes them to be instinctively more cautious about the potential consequences of their cooperative behaviour.

The fact that older participants were more optimistic than younger ones also aligns with previous findings and with the socioemotional selectivity theory which posits that aging is associated with increasing motivation to derive emotional meaning from life, leading to a general positivity bias^52,53^. The fact that older people were found to be more optimistic, promoting their contributions in the PGG as well as their expectation about others’ positive intentions, appears to support the claim that motivations and investments change during aging (with optimism being a mechanism driving such changes).

When analysing the role of conditional cooperation through different informational settings by varying the expected contributions of partners, we found reciprocity and intuition to be the key drivers of cooperative behaviour. More specifically, individuals responded to the information received by contributing more when faced with generous partners, and less when faced with stingy ones. Further, participants in the time-pressure condition contributed more than those in the time-delay condition. Results on conditional cooperation also established that younger individuals were more inclined to “punish” partners who made more heterogeneous contributions (e.g., 4 and 0 lottery tickets), while older individuals focused more on the fact that one partner was cooperating and less on the fact that the other one was defecting. Again, this is in line with a general optimism bias displayed by the older but not by the younger participants.

DG results revealed that altruistic behaviour also appears to be supported by a dual-process cognitive architecture which however influences the development of altruism differently than the development of cooperation. Altruism was found to increase non-linearly with age: first declining from adolescence until adult age, and then increasing rapidly across adulthood and old age. As expected, older people were more generous than younger people, a pattern that has been confirmed across different economic games (for a meta-analysis, see^54^; see also^55–58^). As was the case with cooperation, working-age adults were the group least affected by the time condition and seemed ingrained in their norm of behaviour irrespective of the time constraints.

Lastly, we found that both risk aversion and need to belong partly explain cooperative behaviour. Irrespective of time constraints, more risk-averse individuals cooperated less, and individuals with higher scores in the need to belong scale (measuring the relative desire to be socially accepted) cooperated more, establishing that key behavioural traits shape our cooperative behaviour too^59^. The fact that differences between intuitive and deliberate processes diminished when introducing these factors reveals that both risk aversion and the desire for social inclusion might contribute substantially to the shaping of the social heuristics in cooperative settings.

## Limitations

The study presents limitations that could provide suggestions for future research.

First, for methodological constraints, we were not able to administer the task to individuals younger than 13 years of age. Assessing the same tasks on children would have required a massive change in timing, questions asked, which would have made it difficult to directly compare the results across ages. However, extending the developmental trajectory would provide a broader view on the mechanisms accounting for intuitive vs deliberative cooperation, and provide a stronger test for the hypothesis that experience with cooperative behaviours and contexts is key for how humans tune to automatic responses (i.e., more intuitive cooperation in this case).

Second, in the current study, participants’ responses to how much they thought others contributed or donated were not incentivised. This might have led to more hasty and inaccurate answers or at least less motivated than would be predicted in an incentivised task. For instance, Gächter and Renner^60^ have shown that belief accuracy is higher when responses are incentivised, and incentivised responses promote higher contribution levels in public good games. However, it should be noted that other studies did not use any incentive on beliefs and found nevertheless a causal effect of beliefs on contributions^61^.

Finally, as potential factors influencing cooperation here we considered altruism, risk aversion, and optimism, but we acknowledge that other factors could explain changes in intuitive and deliberative cooperation across the lifespan, such as socio-economic status, occupation, education, as well as what has been termed ‘fitness interdependence’^62^. Indeed, while its original meaning refers to our evolutionary fitness in terms of survival and reproduction (i.e., genetic relatedness), it has been recently enlarged to include relationships that promote individual fitness. These can be marriages, but also military units, and places/situations in which tight and ‘special’ relationships can be formed. In this perspective, it would be important to investigate cooperation strategies across the diversity and complexity of human relationships, from complete strangers to family members, friends and romantic partners^63,64^.

## Conclusions

This study has shown how intuitive processes shape both cooperative and altruistic behaviours differently across the lifespan. Intuition played a limited role in working-age adults in both games (with adults relying on well-established heuristics irrespective of the time condition), but affected all other age groups significantly. Cooperation was shown to grow over the lifespan and was found to be intuitive in young adults and older adults, but to be a deliberative process in adolescence. On the contrary, altruism was shown to decline until adulthood only to increase towards old age and to be intuitive in all age groups except for working-age adults. Expectations about others’ prosocial behaviour, but also personal predispositions or personality traits, all appear to contribute to the shaping of the cooperative humans that we become.

## Methods

### Experimental model and participants details

Participants were recruited by word of mouth across the University of Milano-Bicocca and University of Trento (Italy) and through online fliers and advertisements posted on Facebook and on a few online journals. A total number of 390 Italian speaking individuals took part in the study, but the final analyses included 382 participants, since 8 participants did not report their age and were consequently excluded from the analyses. All participants signed an informed consent prior to testing. For the minors (12–17-year-olds), the parents or legal guardians also signed an informed consent. The sample was divided in four age groups: adolescents (*n* = 98; age range: 12–19 years; *M*_*age*_ = 15.7, *SD* = 2.1; 72 female), young adults (*n* = 122; age range: 20–30 years; *M*_*age*_ = 22.9, *SD* = 2.3; 83 female), adults (*n* = 67; age range: 31–60 years; *M*_*age*_ = 45.4, *SD* = 9.2; 36 female), and older adults (*n* = 95; age range: 61–79 years; *M*_*age*_ = 65.7, *SD* = 5.1; 47 female). This division was made capitalising on brain, cognitive and experiential changes occurring across these life stages. In other words, while there are massive changes occurring in the adolescent years (in terms of both neural and behavioural changes), less changes are observed (or at least are much slower) in adults and during ageing. In particular, around age 13 the massive brain changes that is observed in childhood levels off, while there occurs another wave of growth or at least no tissue loss between 18 and 25 years of age. Moreover, volume loss is observed after age 35, and accelerates around age 60 (^65^; see^66^ for sleep as a marker of the end of adolescence, marked at age 20). More generally, adolescence is commonly considered as spanning between 11 and 19 years of age (^67^, with specific changes occurring in the so-called ‘social brain’^68^, which supports complex cognitive and social capacities, such as the ability to understand others, self-consciousness, emotional control, inhibition and attention skills. It should be noted that the number of individuals per group was kept as comparable as possible to avoid statistical unbalance.

Participants were invited to take part in an online survey and received a link to the study, which was run on Pavlovia (https://pavlovia.org/). Participants were not paid and completed the survey on a voluntary basis. However, they were told that by participating, they had the chance to win one of two 50-euro prizes, which would be assigned at the end of the study. They were also told that the odds of winning 50 euros were calculated on the basis of the number of lottery tickets earned while playing the economic games in the survey (the more tickets earned during the game, the higher the chance of winning the prize). The study was approved by the Ethical committee of the University of Milano-Bicocca, following the guidelines of the Declaration of Helsinki for human research.

### Tasks and procedure

The survey took 25–30 min to complete and comprised two main games: the public goods game (PGG, presented in several versions, with or without prior information about the other players’ contributions) and the dictator game (DG). We also elicited risk aversion (RA), optimism bias (OB) and administered at the end the need to belong scale (NB).

A series of videos displayed before the tasks served to explain participants what to do. Informed consent and signed permission/authorization was obtained from the actor to publish the videos in the manuscript.

#### Public goods game (PGG)

To understand cooperative behaviour, we first ran a one-shot version of the public goods game in which each participant was matched with two other (fictitious) participants. In the game, participants were instructed that both themselves and the other two players would receive four lottery tickets and that they had to decide how many tickets to contribute to a common fund (by entering a number between 0 and 4; for the explanation of the game and the instructions given to participants, see Movie S1). The total amount of tickets placed in the common fund would then be doubled and divided evenly among the three players. The maximal number of tickets (namely, 8) would be obtained when the three partners contributed all their tickets to the common fund. The selfishly optimal strategy was not to contribute any tickets irrespective of the decisions taken by the partners.

About half of the participants were randomly assigned to the time-pressure condition, whereas the other half was assigned to the time-delay condition. In the time-pressure condition, participants had only 10 s to make their decisions and select their answers, whereas in the time delay condition, they were told that they had to wait at least 10 s before providing their answers, and then had 20 s to respond (see in the Extended Data: Basic Data Checks). Thus, whereas in the time-delay condition participants were instructed to take their time (at least 10 s) for reasoning before making their decision, in the time-pressure condition they were instructed to respond quickly by relying on intuition. Instructions given to participants were specifically tailored to either the time-pressure or the time-delay condition (see Movies S2 and S3).

#### Expected and conditional reciprocity

To investigate the expectations of participants about the cooperative behaviour of their fictitious partners, we asked them to estimate how many tickets the other two players contributed, by inserting a number between 0 and 4 for each of their partners. Then, we investigated the tendency of individuals to engage in conditional cooperation by having them play the PGG again under the same time condition, but while presenting them first with the typical contributions for the two anonymous partners (see Supplementary Material and Movies S4 and S5 for details). In particular, the following five informational settings were considered: (1) Partner A = 0, Partner B = 1; (2) Partner A = 2, Partner B = 2; (3) Partner A = 3, Partner B = 4; (4) Partner A = 3, Partner B = 1; (5) Partner A = 0, Partner B = 4.

#### Dictator game (DG)

After completing the PGG, participants played a one-shot Dictator game (see Supplementary Material and Movies S6 and S7 for details). As in the PGG, participants played under two time-conditions (time-pressure and time-delay) and had to decide how many of their four lottery tickets to donate to the other partners, explicitly being informed that they would not have received anything in exchange of their donation.

#### Risk aversion task

Once completed the DG, participants completed a classical risk aversion task^42^ measuring aversion to risk (i.e., the preference for certainty over risky outcomes even when the expected returns are identical, for instance when one prefers earning 50 euros with certainty than gambling to win 100 euros or nothing with a 50% probability). Participants were presented with pairs of boxes and were told that they had to choose one of them. Each box contained a probability distribution over lottery tickets. For instance, participants were asked to choose between Box 1 containing 10 tickets with 50% probability and none otherwise, and Box 2 containing 5 lottery tickets with certainty. The following seven choices were presented to subjects:

- 10 tickets with probability 50% and none otherwise vs 5 tickets for sure.
- 7 tickets for sure vs 10 tickets with probability 50% and none otherwise.
- 3 tickets for sure vs 10 tickets with probability 50% and none otherwise.
- 10 tickets with probability 50% and none otherwise vs 8 tickets for sure.
- 6 tickets for sure vs 10 tickets with probability 50% and none otherwise.
- 10 tickets with probability 50% and none otherwise vs 4 tickets for sure.
- 2 tickets for sure vs 10 tickets with probability 50% and none otherwise.

#### Optimism bias

In the Optimism Bias task, participants were presented with 10 statements depicting the probability of negative events (e.g., “50% of Italian couples divorce at least once in their lifetime”) occurring in the national or worldwide population and were asked to report the likelihood that that same event could happen to them in the future (see Supplementary Material for the list of items).

#### Need to belong

Lastly, participants answered a 10-item questionnaire, known as the *Need to Belong Scale*^43^ aimed at assessing their desire and need for social contact and inclusion (see Supplementary Material for the complete list of items).

### Quantification and statistical analysis

All data were analysed using Stata (https://www.stata.com/). Statistical details of the experiment can be found in the Result section. For the analysis, significance was taken to be 0.05. However, in the tables in the text we also highlighted when p < 0.15 for the reader to be able to distinguish from p > 0.15.

The minimum sample size required to detect an effect size = 0.3 (medium) with an Ordered Probit analysis was calculated a-priori using the following parameters: alpha = 0.05, power = 0.8. Power calculation meant a sample of roughly 350 subjects. Only subjects who did not report age were excluded from all the analyses. In some regressions, subjects had further to be excluded if they did not complete the specific task being analysed, but we had a limited number of instances in which that was the case. The treatment was assigned randomly by the experimenter, who was blind to the characteristics of the participants, except age.

### Extended data

#### Gender, risk aversion, need to belong, and response times within condition

Gender was added to specification (3) as an additional control as follows

13 $$PGG=k+a*Age+b*Time+c*Time*Age\_Group+d*Female$$

where $Female$ is a variable which is equal to 1 for female participants and to 0 for male ones. Table 2 shows that females tended to cooperate slightly more than males.

Elicited risk aversion and a measure of desire for social acceptance were then added to specification (3) as additional controls as follows

14 $$PGG=k+a*Age+b*Time+c*Time*Age\_Group+d*CE+e*NB$$

where $CE$ denotes our elicited measure for certainty equivalents with high values denoting more aversion to risk, and where NB is our elicited measure of desire for social acceptance with high values denoting more desire. Table 2 shows that participants that were more risk averse and/or displayed more need for social acceptance tended to cooperate more.

An additional analysis carried out on the baseline PGG looked at the effects of predicted response times with each time condition (time-pressure vs time-delay). Such analysis consisted of a two-stage regression where predicted response times were first estimated conditional on the time condition and age (through a standard OLS regression), and PGG contributions were estimated using our baseline specification (3) but replacing age with the predicted response times (to avoid collinearity problems), as follows

15 $$PGG=k+a*Predicted\_RT+b*Time+c*Time*Age\_Group$$

where $Predicted\_RT$ denotes the predicted response times. We relied on predicted response times rather than actual response times for this analysis as correlation between actual response times and Probit residuals might have otherwise violated exclusion restrictions. The first stage regression (omitted for sake of brevity here) established that individuals under time-pressure took on average 5.5 s to respond to the PGG task whereas those under time delay took on average 16.8 s to respond, with older individual spending surprisingly slightly less time on the task. The second stage Probit regression then established that within each condition, higher response times lead to significantly lower PGG contributions, as shown in Table 2.

#### Optimism, age, PGG contributions and beliefs

The effect of optimism on PGG contributions and expected cooperation was also analyzed. Simple OLS regressions established that optimism increased both variables as displayed in Table 4. Further, age was shown to be positively associated with optimism as displayed in Table 5.

#### Conditional cooperation

Table 6 summarizes estimates of specification (7) for the five informational settings. When partners’ contributions were low (Resp_0–1 case), participants contributed 61% of what they contributed in the original PGG. In the three settings in which the average partners’ contributions were equal to 2 (Resp_2–2, Resp_0–4, and Resp_3–1 cases), participants contributed 81% of what they contributed in the original PGG. When contributions were high (Resp_3–4 case), participants contributed as in the original PGG. These correlations were estimated by running OLS regressions without constants, having the original contribution as an independent variable and the new contribution as a dependent one.

When the time condition was added to the regressions as in specification (8), time-pressure was shown to consistently increase contributions across the five informational settings as shown in Table 7. For instance, in the Resp_0–1 case, participants under time-delay contributed 57% of their uniformed PGG contribution, while participants under time-pressure contributed 57% of their uninformed PGG contribution plus 0.21 tickets. Table 7 reports similar insights for the five informational settings. Surprisingly, in the time-delay condition participants contributed only 92% of their uninformed contribution even when faced with the most generous partners, contributing respectively 3 and 4 tickets.

Key insights remained unaffected by adding a constant and age as explanatory variables as in specification (9). But age was shown to be positively associated to the PGG contributions in all the five informed setting even condition on uniformed PGG contributions, despite these increasing with age. Such effects were significant only in the Resp_3–1 and Resp_0–4 settings and explained only a minor part of the contributions. More interestingly, adding constants had a profound effect on the estimation by differentiating the three settings in which partners’ average contributions amounted to 2. More equitable contributions by partners (the Resp_2–2 case) were found to have a higher constant and a lower slope than the case in which contributions were most inequitable (the Resp_0–4 case), implying that inequality in partners’ contributions led to more sensitive responses than when partners behaved similarly. These results are reported in detail in Table 8.

For each participant, the variance of PGG contributions across the five informational settings was then computed to derive a measure of responsiveness to information. Such a variable, called responsiveness, was constructed by computing the empirical variance of the Resp_0–1, Resp_2–2, Resp_3–4, Resp_0–4 and Resp_3–1 variables for each participant meaning that the contributions of participants with a higher responsiveness varied more across informational cases. Responsiveness was then shown to be negatively associated with age, through an OLS regression, with age and a constant as a regressors and responsiveness as a dependent variable (with results reported in Table 11). Older participants were shown to be less responsive to information relative to younger ones.

**Table 11:** OLS estimates with responsiveness as dependent variable and age and a constant as regressors.

Variables	Responsiveness
Constant	0.77***
Age	− 0.003*

*p < 0.05, ***p < 0.001.

When responsiveness was added to the main Probit specification (3) it was shown to increase PGG contributions as displayed by the ordered Probit regression in Table 12, meaning that participants whose contributions varied more across informational settings were those who had contributed more in the original uniformed PGG.

**Table 12:** Ordered probit estimates for specification (3) with responsiveness as an additional regressor.

Variables	PGG
Time	− 1.31*
Age	0.03**
Responsiveness	0.14^+^
Slow × adolescent	(Baseline)
Slow × early adult	− 0.53**
Slow × adult	− 0.71^+^
Slow × older adult	− 1.77**
Fast × adolescent	1.04^+^
Fast × early adult	1.00*
Fast × adult	0.55^+^
Fast × older adult	(Omitted)
Contribute 0.5	− 1.46***
Contribute 1.5	0.36
Contribute 2.5	0.61*
Contribute 3.5	1.58***
Participants	(352)

^+^p < 0.15 (marginally significant), *p < 0.05, **p < 0.01, ***p < 0.001.

To conclude the analysis of conditional cooperation, we report results for our main specification (3) but with the dependent variable, PGG, replaced by the informed contributions, Resp_x–y, to display the effect of age, time condition, and their interaction on conditional cooperation (see Table 13). While the effect of age on contributions is consistent across the five informed settings, the effect of the time condition differs considerably across these and across age groups. Further, variability of parameter estimates was considerably increases in some of the informational settings. This is particularly evident in the informational case in which partners’ contributions were low since several participants reduced their contributions considerably whereas others did not, making parameter estimates less precise.

**Table 13:** Ordered probit specification (3) but with informed contributions as dependent variables.

Variables	Resp_0-1	Resp_2-2	Resp_3-4	Resp_0-4	Resp_3-1
Time	− 0.91^+^	− 0.81^+^	− 0.54	− 1.28*	− 0.84^+^
Age	0.02*	0.02*	0.02^+^	0.03**	0.02*
Slow × Adolescent	(Baseline)	(Baseline)	(Baseline)	(Baseline)	(Baseline)
Slow × early adult	− 0.65**	− 0.18	0.01	− 0.33^+^	− 0.14
Slow × adult	− 0.72^+^	− 0.26	− 0.19	− 0.27	− 0.29
Slow × older adult	1.34*	− 0.95^+^	− 0.86^+^	− 1.28*	− 0.93^+^
Fast × adolescent	1.03^+^	1.07^+^	0.69	1.32*	0.76
Fast × early adult	0.42	0.79^+^	0.43	1.37**	1.00*
Fast × adult	0.23	0.41^+^	0.63*	0.95**	0.80**
Fast × older adult	(Omitted)	(Omitted)	(Omitted)	(Omitted)	(Omitted)
Contribute 0.5	− 0.69**	− 1.29***	− 1.22***	− 0.49*	1.09***
Contribute 1.5	0.32	− 0.22	− 0.47***	0.07	− 0.14
Contribute 2.5	1.29***	1.12***	0.17	1.17***	1.17***
Contribute 3.5	1.76***	1.73***	1.01***	1.77***	1.85***
Participants	(365)	(370)	(369)	(366)	(371)

^+^p < 0.15 (marginally significant), *p < 0.05, **p < 0.01, ***p < 0.001.

#### Dictator game

Table 12 reports results for the dictator game of the three key Probit specifications discussed in the main text. In contrast to the PGG, time-pressure had a more uniform effect on DG donations. Time-pressure raised DG donations consistently across age groups (except for adults for whom contributions were not affected by the time condition). Thus, intuitive reasoning consistently led to more altruism than reasoned choices supporting the view that altruism is an intuitive and deeply rooted trait of human behaviour (with the usual caveat, that in working-age adults intuitive and deliberative reasoning seem to coincide).

To understand the effect of altruism on PGG contributions, we then analysed two of our baseline specifications, but with DG donations added as a regressor, namely:

16 $$PGG=k+a*Age+b*Time+c*Time*Age\_Group+d*DG$$

17 $$PGG=k+a*Age+b*Time+c*Time*Age\_Group+d*DG+e*CE+f*NB$$

When DG donations were added as a regressor to these specifications, donations became the main variable explaining PGG contributions dampening the effects of the other variables, as can be seen in Table 10. But these specifications also highlight a close association between cooperation and altruism, as measured in the PGG and DG.

#### Basic data checks

The following checks display key features of the sample and the data. Table 14 shows how the sample is split across the four age groups used in much of our empirical specifications. The sample of young adults is slightly larger than the rest, whereas the sample of adults is slightly smaller. The table also highlights that below age 30 the sample included significantly more female participants than male ones. Average age within age groups is also reported in the table. In the young adults group (those aged between 20 and 30) average age was only 22.9 revealing some skewness in the age distribution. Among older adults (those aged between 60 and 80) average age was 65.7 again revealing that our sample had more participants in their sixties than in their seventies.

**Table 14:** Age group frequency and number of participants.

Age group	Participants	Frequency	Mean age	St dev age	Female
Adolescent	98	25.1%	15.7	2.1	72
Young adult	122	31.3%	22.9	2.3	83
Adult	67	17.2%	45.4	9.2	36
Older adult	95	24.4%	65.7	5.1	47
Age not reported	8	2%			

Table 15 establishes that the sample was evenly split between time-pressure and time-delay groups. Further, it documents compliance with the time-pressure treatment by reporting response times quantile by quantile in both the time-pressure and the time-delay conditions. Only 7% of those in the time-pressure group did not comply with the treatment by taking more than 10 s to respond. Further, quantile by quantile, those in the time-pressure group took at least 10 s less to respond than those in the time-delay group. The slowest responders in the time-pressure group took on average 14 s less to respond than those in the same quantile of the time-delay group. Because of this, participants in the time-pressure group taking more than 10 s to respond were retained in the analysis. But results would not be affected by dropping them from the analysis.

**Table 15:** Number of participants and response times by quantile in time-delay and time-pressure groups.

Condition	Participants	1%	5%	10%	25%	50%	75%	90%	95%	99%
Time-delay	186	11.3	11.6	11.8	12.5	13.7	16.4	21.6	26.9	57.7
Time-pressure	191	1.2	1.5	1.7	2.1	2.8	4.5	7.9	16.1	43.2

## Supplementary Information

Supplementary Information 1.

Supplementary Information 2.

Supplementary Information 3.

Supplementary Movie S1.

Supplementary Movie S2.

Supplementary Movie S3.

Supplementary Movie S4.

Supplementary Movie S5.

Supplementary Movie S6.

Supplementary Movie S7.

## Data Availability

The datasets originated and analysed during the current study are available in the OSF repository: https://osf.io/r3dpz/?view_only=80df8756214345e78214703aee3b0029.
