# Supplementary material for: Age-dependent changes in intuitive and deliberative cooperation
Source: Sci Rep. 2023 Mar 17;13:4457. doi: 10.1038/s41598-023-31691-9 (PMC10023788; doi:10.1038/s41598-023-31691-9)
Supplement: Supplementary file 1 — Supplementary Information 1. [file 41598_2023_31691_MOESM1_ESM.docx]

# Supplementary Material

**Age-dependent Changes in Intuitive and Deliberative Cooperation**

# Description of Tasks

The games presented to participants are detailed here below. Both the public goods game and the dictator game were introduced by brief movies, in which an experimenter explained the payoff matrix of the game, providing examples and giving instructions on how to play and respond. Movies containing the instructions given to participants can be downloaded at the following link: <https://osf.io/r3dpz/?view_only=80df8756214345e78214703aee3b0029>.

- Movie S1: General instructions and example of how to play the PGG
- Movie S2: Instructions on how to play the PGG under time pressure
- Movie S3: Instructions on how to play the PGG under time delay
- Movie S4: Instructions on how to play the PGG with prior information about the partners’ choices under time pressure
- Movie S5: Instructions on how to play the PGG with prior information about the partners’ choices under time delay
- Movie S6: Instructions on how to play the DG under time pressure
- Movie S7: Instructions on how to play the DG under time delay

# Public Goods Game (PGG)

We first run a one-shot version of the game, in which participants played with two other (fictitious) participants. Participants were instructed that both herself and the other two players would receive four lottery tickets and had to decide how many tickets to contribute to a common fund (see Movie S1). The total amount of tickets placed in the common fund would then be doubled and divided evenly among the three players. Thus, the maximum payoff for each individual is reached if all players contribute all four tickets.

Instructions given to participants were specifically tailored to either the time pressure or the time delay condition (see Movie S2 and S3). Indeed, about half of the participants were assigned to the time pressure condition, whereas the other half was assigned to the time delay condition (see main text in the article).

Before starting the game, participants read the consent form and agreed to participate by pressing on the space bar of their computer. Following consent, participants saw another screen, in which they were instructed on how to provide their response, which consisted in selecting a number from 0 to 10 using the mouse cursor along a line, followed by a space bar press to confirm the answer. The exact text with the instructions given to the participants is provided below.

Before we start, it is important that you familiarize with the type of answer you can provide. In all the games, you will be asked to insert a numeric answer that you can select by placing the mouse cursor along a line like the one you see at the bottom of the screen. Select a number by placing the mouse cursor along the line, and then press the space bar to confirm your answer.

Once inserted their answer and confirmed their choice, participants were presented with another screen, in which they were instructed how to respond under time pressure or time delay. Participants assigned to the time pressure condition were invited to insert an answer within 10 seconds, during which a visual countdown appeared on the screen to keep track of the time. In the time delay condition, participants were invited to insert an answer after having waited for 10 seconds. Again, a 10-second countdown appeared, but participants could not provide any answer during this period.

[Time pressure] Great! Now try to insert a number between 1 and 10 within 10 seconds. When you press the space bar, a countdown will start that will help you to keep track of the time you have to provide your answer. Remember to press the space bar once you have selected your answer!

[Time delay] Now try to insert a number between 1 and 10 after having waited for 10 seconds. When you press the space bar, a countdown will start that will help you keeping track of the time you have to wait before you can provide your answer. Please remember to press the space bar once you have selected your answer!

Next, participants were presented with a 100s movie (Movie S1), in which a female experimenter gave them a set of instructions regarding the one-shot PGG. Specifically, they were told that they would interact with two anonymous players, and that each of them would receive four lottery tickets. Next, they learned that they could choose how many tickets to contribute to the common fund (one, two, three, four, or none) and that once in the common fund, the overall amount of tickets given by all participants would double and then redistributed equally among the three players. The exact text with the instructions given to the participants in Movie S1 is provided here below, translated in English and in its original Italian version. To help participants understand the game, in the movie the experimenter also provides an example, by asking participants to imagine that all participants contributed two tickets and explaining how they would end up with six tickets each.

Movie_S1 [The experimenter is sitting at a table facing the camera, and in front of her there are four squared slips of paper representing the four lottery tickets initially given to each participant] Hello! And thank you for having decided to participate in this research! In the next 30 minutes, you will be presented with a series of tasks that do not aim to challenge you or assess your cognitive abilities. There are no right or wrong answers. Remember to answer in the most spontaneous way. Let’s start the game! In this game, other two persons will participate together with you. Each participant starts off with four lottery tickets. [The experimenter points to the four slips of paper in front of her]. With these tickets, it is possible to win one of the three 50-euro lottery prize. The more tickets you earn during the game, the higher the chance of winning a cash prize. The game requires you to choose how many tickets [the experimenter points to the slips] you want to contribute to the common fund. You can decide to invest one ticket, two tickets, three tickets, all the four tickets or none of them. Once contributed to the common fund [the experimenter, while talking, takes with her left hand a rectangular basket on which can be read ‘common fund’; then places it in the centre of the table, in front of her], the tickets you and the other two participants invested will double and be redistributed equally among all the participants. Let’s make an example to understand better. If all the participants, you included, decide to contribute two tickets out of four [the experimenter inserts two slips in the common fund/rectangular basket, followed by the other two virtual participants, who place two slips each into the common fund], for a total of six tickets, once inserted in the common fund, they duplicate and become 12. These 12 tickets would then be redistributed equally among all participants, so that each participant receives four tickets, which, added to the ones you did not invest, become six in total.

Right after the end of Movie_S1, participants were taken to a Review-Instructions Screen, in which they could read a four-point list with the instruction of the PGG:

Let us go through the game instructions once more: 1) The tickets you decide to contribute are doubled in the common fund; 2) Then, all the tickets in the common fund, the ones you and the other participants invested, are divided equally among the participants; 3) Remember that the tickets obtained from the common fund must be added to the tickets that you did not invest; 4) Finally, remember that you do NOT know how much the other participants will contribute. Press the space bar to continue.

Next, in a second movie, participants received other instructions to play the PGG, given again by the same female experiment of Movie S1. In the time pressure condition, the experimenter told in the 27s movie that participants had only 10 seconds to decide and respond how many tickets to contribute (see Movie S2). Instead, in the time delay condition, the experimenter asked participants in a 31 s movie to think carefully about their decision and told them to wait 10 seconds before answering, after which they had 20 seconds to provide their answer (see Movie S3). The exact text with the instructions given to participants in Movies S2 and S3 is provided here below.

Movie_S2_Time_Pressure [The experimenter is sitting at a table facing the camera, and in front of her there are four squared slips of paper representing the four lottery tickets initially given to each participant] Perfect, now the game starts. You have four lottery tickets, they are yours. As soon as the countdown starts, you will have only 10 seconds to decide how many of these four tickets you want to contribute to the common fund. Please select a number from 0 to 4 by moving the mouse cursor along the line that will appear at the bottom of the screen. Please remember that you have only 10 seconds, thus respond as spontaneously as possible. Are you ready? At the end of the video the countdown starts.

Movie_S3_Time Delay [The experimenter is sitting at a table facing the camera, and in front of her there are four squared slips of paper representing the four lottery tickets initially given to each participant] Perfect, now the game starts. You have four lottery tickets, they are yours. Please, think carefully before taking a decision. You have to wait 10 seconds before entering your answer. You will see a countdown notifying you when these 10 seconds have passed. Next, you will have 20 seconds to enter your answer. You will have to select a number from 0 to 4 by moving the mouse cursor along the line that will appear at the bottom of the screen. Are you ready? At the end of the video the countdown starts.

As soon as the movie ended, the 10s countdown started, and participants had to insert a number between 0 and 4 within this time window in the time pressure condition or wait 10 seconds before answering in the time delay condition.

# Expected Reciprocity

Once participants inserted their answer, they were asked to estimate how many tickets the other two players contributed. Participants’ instructions on the screen were as follows: “According to you, how much did the other two participants contribute? Please insert the two amounts (between 0 and 4) below ‘PARTNER A’ and ‘PARTNER B’, respectively. To continue, press the space bar.” No time constraints were inserted here.

# Conditional Cooperation

Once participants provided their answers and pressed the space bar, they saw another movie (Movie S4 and S5), in which the experimenter explained the rules of a different version of the PGG. In this version of the game, participants were informed about the typical contribution of the other two anonymous participants (defined as the common contribution of the two partners). Instructions given to participants in 16s Movie S4 (Time Pressure) and in the 38s Movie S5 (Time Delay) are provided here below.

Movie_S4_Time_Pressure [Stage/background as before] Now you will play again but with two new participants. However, this time we will tell you how much the other two participants commonly contribute. As before, you will have only 10 seconds to enter your answer. Are you ready? At the end of the video the countdown starts.

Movie_S5_Time_Delay Now you will play again but with two new participants. However, this time we will tell you how much the other two participants commonly contribute. As before, once the countdown starts, you have 10 seconds to decide how many of these tickets you want to contribute to the common fund. Once these 10 seconds have passed, you will have 20 more seconds to enter your answer. Are you ready? At the end of the video the countdown starts.

Participants played five rounds, each with a different pair of anonymous players. On each round, participants read: “This is the common behaviour of the participants you have to play with now: PARTNER A: X tickets; PARTNER B: Y tickets. How much do you contribute?”

Here below the number of tickets commonly contributed by Partner A and Partner B on each round of the game:

- Round 1: Partner A = 0, Partner B = 1
- Round 2: Partner A = 2, Partner B = 2
- Round 3: Partner A = 3, Partner B = 4
- Round 4: Partner A = 3, Partner B = 1
- Round 5: Partner A = 0, Partner B = 4

Lastly, to make sure that participants really understood the payoff structure of the game, participants answered a comprehension probe on a new screen. In particular, they read the following question and had to choose which of the three given options was the correct one (i.e., Number 1).

Attention! Now you have to answer a COMPREHENSION PROBE about the games you just completed: What happened to the lottery tickets once they were inserted into the common fund? Please select the correct answer: 1. they doubled and then they were divided equally among all the three participants; 2. they quintupled and then they were all given to the other two participants and none to you; 3. they quintupled and then were divided equally among all the three participants.

# Dictator Game (DG)

After completing the last round of the PGG, participants were taken to a new screen and presented with a movie (Movie S6 and S7), in which the experimenter provided the instructions for DG. Here again, participants played under two time-conditions (time pressure and time delay), and the instructions given to participants in the 33s Movie S6 (Time Pressure) and in the 39s Movie S7 (Time Delay) are provided here below.

The dictator game is widely used in psychology to assess pure altruism (4). If participants play the role of ‘dictators’, they are asked to divide a certain amount of money or resources given to them between themselves and one anonymous recipient. Here participants were instructed that they would have played a game like the one they played before (i.e., the public goods game), with the exception that now the lottery tickets they decided to contribute would have been inserted in the common fund and then divided equally among the other two participants only, thus without the possibility of receiving anything in return. With respect to the time given for providing an answer, the same rules and instructions of the time pressure and time delay conditions in the public goods game applied here.

Movie_S6_Time_Pressure In this game, you will play with two new participants. Again, each player receives four lottery tickets and can decide whether contributing with one, two, three, all the four tickets or none of them. However, this time you will receive nothing in return. Thus, what you decide to contribute, if you decide to contribute anything, will be distributed equally among the other two participants. Again, you will only have 10 seconds to decide how many tickets you want to contribute. Are you ready? At the end of the video the countdown starts.

Movie_S7_Time_Delay In this game, you will play with two new participants. Again, each player receives four lottery tickets and can decide whether contributing with one, two, three, all the four tickets or none of them. However, this time you will receive nothing in return. Thus, what you decide to contribute, if you decide to contribute anything, will be distributed equally among the other two participants. You will have 10 seconds to decide how many of these tickets you want to contribute to the common fund. Please, think carefully about your decision during the 10 seconds you have before answering. Are you ready? At the end of the video the countdown starts.

# Risk Aversion Task

Once completed the DG, participants played the Risk Aversion Instructions Screen where they could read a brief text explaining how the new task worked. The risk aversion task measures aversion to risk and uncertainty (5), that is, the preference for certainty over risk even when the expected outcomes are identical, for instance when one prefers earning 50 euros with probability 1 (certainty) than gambling to win 100 euros or nothing with a 0.5 probability.

Here participants read that to start off with this game they received 20 new lottery tickets. Next, they would have had the task to choose between two options or boxes. Each box would have been associated with a different probability to win or lose a certain number of lottery tickets. Specifically, participants read:

To take part in this game, we give you 20 lottery tickets. In this game, you will have to choose between two boxes. Please read carefully what they both contain. You do not have to provide an answer within a certain time limit, but please try to be as spontaneous and quick as possible. Press the space bar to continue.

Once participants pressed the space bar, they were presented with a series of trials, in which pairs of boxes appeared. The text explaining the content of the two boxes was as follows, “If you choose BOX 1, you have 50% probability to win X lottery tickets, while if you choose BOX 2, you will certainly win Y lottery tickets. Which box do you choose? Select your answer here below”. Four out of seven trials presented first the box with probability 1, whereas the other three trials described the box with probability 1 as second. Moreover, in the first trial, the text specified that a 50% probability of winning X lottery tickets corresponded to a 50% probability of losing X lottery tickets. Here below the number of tickets associated with each box for each of the seven trials that were administered to participants.

- Trial 1: BOX 1 = 50% 10 tickets, BOX 2 = 100% 5 tickets
- Trial 2: BOX 1 = 100% 7 tickets, BOX 2 = 50% 10 tickets
- Trial 3: BOX 1 = 100% 3 tickets, BOX 2 = 50% 10 tickets
- Trial 4: BOX 1 = 50% 10 tickets, BOX 2 = 100% 8 tickets
- Trial 5: BOX 1 = 100% 6 tickets, BOX 2 = 50% 10 tickets
- Trial 6: BOX 1 = 50% 10 tickets, BOX 2 = 100% 4 tickets
- Trial 7: BOX 1 = 100% 2 tickets, BOX 2 = 50% 10 tickets

**Optimistic Bias**

We developed three different versions of the scale, one of each of the following three age groups: adolescents (13-19 years), young adults and adults (20-59 years), older adults (60-80 years). Here below we provide the full list of items for each age group. For each item, participants were asked, “What is the probability that this event might happen to you in the future?”

Items used with adolescents:

- 40% of people lose one of their two grandfathers before they turn 20)

- 30% of people lose an important friend due to a fight at least once before the age of 20

- 30% of 30-40-year-olds fail to have children

- 20% of students fail their final exams

- 50% of people miss at least one flight before the age of 30

- 20% of people go deaf before age 60

- 80% of people are left by their boyfriend/girlfriend at least once before the age of 20

- 80% of people have a car accident before the age of 30

- 50% of people have their mobile phone stolen at least once before age 25

- 70% of people fail their driving test at least once before the age of 20

Items used with young adults and adults:

- 45% of *** (blinded for reviewing purposes) have been betrayed by their partner

- 30% of *** (blinded for reviewing purposes) have had their credit card cloned

- 30% of the world population develops cancer

- 20% of the world population have had drug problems

- 50% of the *** (blinded for reviewing purposes) dies before 85 years of age

- 20% of the *** (blinded for reviewing purposes) suffers from infertility

- 70% of the *** (blinded for reviewing purposes)experienced being fired

- 80% of the *** (blinded for reviewing purposes) has missed a flight

- 80% of the *** (blinded for reviewing purposes) develops hearing loss (deafness) after the age of 70

- 50% of *** (blinded for reviewing purposes) couples divorce at least once in their lifetime

Items used with older adults:

- 45% of the *** (blinded for reviewing purposes) have witnessed an accident happened to a dear one

- 30% of the *** (blinded for reviewing purposes) have their credit card cloned

- 30% of the world population develops cancer

- 20% of the world population experienced falling once from the stairs

- 50% of the *** (blinded for reviewing purposes) population had a panic attack at least once before age 80)

- 20% of the world population suffered a theft in their home

- 50% of the *** (blinded for reviewing purposes) dies leaving debts to their families

- 20% of the *** (blinded for reviewing purposes) receives cataract surgery after age 80

- 20% of the *** (blinded for reviewing purposes) develops dementia after age 80

- 40% of the *** (blinded for reviewing purposes) receives a telephone scam at least once in their life)

**Need to Belong**

To assess subjective desire for social contact, inclusion, and social acceptance, we used the 10-item survey developed by Leary et al. (2013).

-If other people don’t seem to accept me, I don’t let it bother me

-I try hard not to do things that will make other people avoid or reject me

-I seldom worry about whether other people care about me

-I need to feel that there are people I can turn to in times of need

-I want other people to accept me

-I do not like being alone

-Being apart from my friends for long periods of time does not bother me

-I have a strong “need to belong”

-It bothers me a great deal when I am not included in other people’s plans

-My feelings are easily hurt when I feel that others do not accept me
