# Supplementary material for: Age-dependent changes in intuitive and deliberative cooperation
Source: Sci Rep. 2023 Mar 17;13:4457. doi: 10.1038/s41598-023-31691-9 (PMC10023788; doi:10.1038/s41598-023-31691-9)
Supplement: Supplementary file 2 — Supplementary Information 2. [file 41598_2023_31691_MOESM2_ESM.pdf]

Extended Data

Age-dependent Changes in Intuitive and Deliberative Cooperation

Gender, Risk Aversion, Need to Belong, and Response Times within Condition

Gender was added to specification (iii) as an additional control as follows

$$PGG = k + a * Age + b * Time + c * Time * Age\_Group + d * Female \quad \text{(xiii)}$$

where $Female$ is a variable which is equal to 1 for female participants and to 0 for male ones. Table E1 shows that females tended to cooperate slightly more than males.

Elicited risk aversion and a measure of desire for social acceptance were then added to specification (iii) as additional controls as follows

$$PGG = k + a * Age + b * Time + c * Time * Age\_Group + d * CE + e * NB \quad \text{(xiv)}$$

where CE denotes our elicited measure for certainty equivalents with high values denoting more aversion to risk, and where NB is our elicited measure of desire for social acceptance with high values denoting more desire. Table E1 shows that participants that were more risk averse and/or displayed more need for social acceptance tended to cooperate more.

An additional analysis carried out on the baseline PGG looked at the effects of predicted response times with each time condition (time-pressure vs time-delay). Such analysis consisted of a two-stage regression where predicted response times were first estimated conditional on the time condition and age (through a standard OLS regression), and PGG contributions were estimated using our baseline specification (iii) but replacing age with the predicted response times (to avoid collinearity problems), as follows

$$PGG = k + a * Predicted\_RT + b * Time + c * Time * Age\_Group \quad \text{(xv)}$$

where $Predicted\_RT$ denotes the predicted response times. We relied on predicted response times rather than actual response times for this analysis as correlation between actual response times and Probit residuals might have otherwise violated exclusion restrictions. The first stage regression (omitted for sake of brevity here) established that individuals under time-pressure took on average

5.5 seconds to respond to the PGG task whereas those under time delay took on average 16.8 seconds to respond, with older individual spending surprisingly slightly less time on the task. The second stage Probit regression then established that within each condition, higher response times lead to significantly lower PGG contributions, as shown in Table E1.

Table E1

Ordered Probit Estimates of Specifications(xiii), (xiv), and (xv)

Variables	Specification (xiii)	Specification (xiv)	Specification (xv)
Age	0.03**	0.03*	-
Female	0.22*	-	-
Certainty Equivalent	-	0.06*	-
Need to Belong	-	0.17^{+}	-
Predicted Response Time	-	-	-2.36**
Time	-1.30*	-1.00^{+}	-28.2**
Slow*Adolescent	(baseline)	(baseline)	(baseline)
Slow*Early Adult	-0.47*	-0.46*	-0.47*
Slow*Adult	-0.66^{+}	-0.46	-0.68^{+}
Slow*Older Adult	1.63**	-1.36*	-1.65**
Fast*Adolescent	1.08^{+}	0.76	1.14*
Fast*Early Adult	1.01*	0.81^{+}	1.06*
Fast*Adult	0.54^{+}	0.34	0.57^{+}
Fast*Older Adult	(omitted)	(omitted)	(omitted)
Contribute 0.5	-1.41***	-0.83^{+}	-41.3***
Contribute 1.5	-0.33	0.25	-40.3***
Contribute 2.5	0.65*	1.23**	-39.3***
Contribute 3.5	1.60***	2.20***	-38.3***
Participants	(367)	(339)	(369)

Note. $^{+}$ p < .15, * p < .05, ** p < .01, *** p < .001.

Optimism, Age, PGG Contributions and Beliefs

The effect of optimism on PGG contributions and expected cooperation was also analyzed. Simple OLS regressions established that optimism increased both variables as displayed in Table E2. Further, age was shown to be positively associated with optimism as displayed in Table E3.

Table E2

OLS Estimates with PGG Contributions and Expected Cooperation as Dependent Variables and Optimism and a Constant as Regressors

Variables	Expected Cooperation	PGG
Constant	2.55***	2.90***
Optimism	-0.010**	-0.011**

Note. ** p < .01, *** p < .001.

Table E3

OLS Estimates with Optimism as Dependent Variable and Age and a Constant as Regressors

Variables	Optimism
Constant	46.0***
Age	-0.18***

Note. *** p < .001.

Conditional Cooperation

Table E4 summarizes estimates of specification (vii) for the five informational settings. When partners' contributions were low (Resp_0-1 case), participants contributed 61% of what they contributed in the original PGG. In the three settings in which the average partners' contributions were equal to 2 (Resp_2-2, Resp_0-4, and Resp_3-1 cases), participants contributed 81% of what they contributed in the original PGG. When contributions were high (Resp_3-4 case), participants contributed as in the original PGG. These correlations were estimated by running OLS regressions without constants, having the original contribution as an independent variable and the new contribution as a dependent one.

Table E4

OLS Estimates for Specification (vii)

Variables	Resp_0-1	Resp_2-2	Resp_3-4	Resp_0-4	Resp_3-1
PGG	0.61***	0.81***	1.00***	0.81***	0.81***

Note. Resp_0-1 describes contributions after being told that partner A contributed 0 tickets, and that B contributed 1 ticket. Resp_2-2 describes contributions after being told that both partners contributed 2 tickets. Resp_3-4 describes contributions after being told that partner A contributed 3 tickets and that B contributed 4 tickets. Resp_0-4 describes contributions after being told that partner A contributed 0 tickets and that B contributed 4 tickets. Resp_3-1 describes contributions after being told that partner A contributed 3 tickets and that B contributed 1 ticket.

*** $p < .001$.

When the time condition was added to the regressions as in specification (viii), time-pressure was shown to consistently increase contributions across the five informational settings as shown in Table E5. For instance, in the Resp_0-1 case, participants under time-delay contributed 57% of their uniformed PGG contribution, while participants under time-pressure contributed 57% of their uninformed PGG contribution plus 0.21 tickets. Table E5 reports similar insights for the five informational settings. Surprisingly, in the time-delay condition participants contributed only 92% of their uninformed contribution even when faced with the most generous partners, contributing respectively 3 and 4 tickets.

Table E5

OLS Estimates for Specification (iix)

Variables	Resp_0-1	Resp_2-2	Resp_3-4	Resp_0-4	Resp_3-1
Time	0.21*	0.34***	0.43***	0.31**	0.39***
PGG	0.57***	0.75***	0.92***	0.76***	0.74***

Note. * $p < .05$, ** $p < .01$, *** $p < .001$.

Key insights remained unaffected by adding a constant and age as explanatory variables as in specification (ix). But age was shown to be positively associated to the PGG contributions in all the five informed setting even condition on uniformed PGG contributions, despite these increasing with age. Such effects were significant only in the Resp_3-1 and Resp_0-4 settings and explained only a minor part of the contributions. More interestingly, adding constants had a profound effect on the estimation by differentiating the three settings in which partners' average contributions amounted to 2. More equitable contributions by partners (the Resp_2-2 case) were found to have a higher constant and a lower slope than the case in which contributions were most inequitable (the Resp_0-4 case), implying that inequality in partners' contributions led to more sensitive responses than when partners behaved similarly. These results are reported in detail in Table E6.

Table E6

OLS Estimates for Specifications (ix)

Variables	Resp_0-1	Resp_2-2	Resp_3-4	Resp_0-4	Resp_3-1
Constant	0.31^{+}	1.04***	1.45***	0.62***	0.96***
PGG	0.46***	0.42***	0.47***	0.51***	0.40***
Age	0.004	0.002	0.001	0.007*	0.006*

Note. $^{+}$ p < .15, * p < .05, ** p < .01, *** p < .001.

For each participant, the variance of PGG contributions across the five informational settings was then computed to derive a measure of responsiveness to information. Such a variable, called responsiveness, was constructed by computing the empirical variance of the Resp_0-1, Resp_2-2, Resp_3-4, Resp_0-4 and Resp_3-1 variables for each participant meaning that the contributions of participants with a higher responsiveness varied more across informational cases. Responsiveness was then shown to be negatively associated with age, through an OLS regression, with age and a constant as a regressors and responsiveness as a dependent variable (with results reported in Table E7). Older participants were shown to be less responsive to information relative to younger ones.

Table E7

OLS Estimates with Responsiveness as Dependent Variable and Age and a Constant as Regressors

Variables	Responsiveness
Constant	0.77***
Age	-0.003*

Note. * $p < .05$, *** $p < .001$.

When responsiveness was added to the main Probit specification (iii) it was shown to increase PGG contributions as displayed by the ordered Probit regression in Table E8, meaning that participants whose contributions varied more across informational settings were those who had contributed more in the original uniformed PGG.

Table E8

Ordered Probit Estimates for Specification (iii) with Responsiveness as an Additional Regressor

Variables	PGG
Time	-1.31*
Age	0.03**
Responsiveness	0.14^{+}
Slow*Adolescent	(baseline)
Slow*Early Adult	-0.53**
Slow*Adult	-0.71^{+}
Slow*Older Adult	-1.77**
Fast*Adolescent	1.04^{+}
Fast*Early Adult	1.00*
Fast*Adult	0.55^{+}
Fast*Older Adult	(omitted)
Contribute 0.5	-1.46***
Contribute 1.5	0.36
Contribute 2.5	0.61*
Contribute 3.5	1.58***
Participants	(352)

Note. $^{+}$ $p < .15$, * $p < .05$, ** $p < .01$, *** $p < .001$.

To conclude the analysis of conditional cooperation, we report results for our main specification (iii) but with the dependent variable, PGG, replaced by the informed contributions, Resp_x-y, to display the effect of age, time condition, and their interaction on conditional cooperation (see Table E9). While the effect of age on contributions is consistent across the five informed settings, the effect of the time condition differs considerably across these and across age groups. Further, variability of parameter estimates was considerably increases in some of the informational settings. This is particularly evident in the informational case in which partners' contributions were low since several participants reduced their contributions considerably whereas others did not, making parameter estimates less precise.

Table E9

Ordered Probit Specification (iii) but with Informed Contributions as Dependent Variables

Variables	Resp_0-1	Resp_2-2	Resp_3-4	Resp_0-4	Resp_3-1
Time	-0.91^{+}	-0.81^{+}	-0.54	-1.28*	-0.84^{+}
Age	0.02*	0.02*	0.02^{+}	0.03**	0.02*
Slow*Adolescent	(baseline)	(baseline)	(baseline)	(baseline)	(baseline)
Slow*Early Adult	-0.65**	-0.18	0.01	-0.33^{+}	-0.14
Slow*Adult	-0.72^{+}	-0.26	-0.19	-0.27	-0.29
Slow*Older Adult	1.34*	-0.95^{+}	-0.86^{+}	-1.28*	-0.93^{+}
Fast*Adolescent	1.03^{+}	1.07^{+}	0.69	1.32*	0.76
Fast*Early Adult	0.42	0.79^{+}	0.43	1.37**	1.00*
Fast*Adult	0.23	0.41^{+}	0.63*	0.95**	0.80**
Fast*Older Adult	(omitted)	(omitted)	(omitted)	(omitted)	(omitted)
Contribute 0.5	-0.69**	-1.29***	-1.22***	-0.49*	1.09***
Contribute 1.5	0.32	-0.22	-0.47***	0.07	-0.14
Contribute 2.5	1.29***	1.12***	0.17	1.17***	1.17***
Contribute 3.5	1.76***	1.73***	1.01***	1.77***	1.85***
Participants	(365)	(370)	(369)	(366)	(371)

Note. $^{+}$ p < .15, * p < .05, ** p < .01, *** p < .001.

Dictator Game

Table 10 reports results for the dictator game of the three key Probit specifications discussed in the main text. In contrast to the PGG, time-pressure had a more uniform effect on DG donations. Time-pressure raised DG donations consistently across age groups (except for adults for whom contributions were not affected by the time condition). Thus, intuitive reasoning consistently led to more altruism than reasoned choices supporting the view that altruism is an intuitive and deeply rooted trait of human behaviour (with the usual caveat, that in working-age adults intuitive and deliberative reasoning seem to coincide).

Table E10

Ordered Probit Estimates for Specifications (x), (xi), and (xii)

Variables	Specification (x)	Specification (xi)	Specification (xii)
Time	0.17	0.57**	-1.32*
Age	0.01*	-	0.04***
Time condition*Age	0.00	-	-
Slow*Adolescent	-	(baseline)	(baseline)
Slow*Early Adult	-	-0.35+	-0.64**
Slow*Adult	-	-0.17	-1.25**
Slow*Older Adult	-	0.22	-1.75**
Fast*Adolescent	-	-0.29*	1.61**
Fast*Early Adult	-	-0.73***	0.90+
Fast*Adult	-	-0.76**	-0.10
Fast*Older Adult	-	(omitted)	(omitted)
Donate 0.5	-0.23	-0.63***	-0.03
Donate 1.5	0.59***	0.21	0.81***
Donate 2.5	1.49***	1.13***	1.76***
Donate 3.5	2.00***	1.66***	2.28***
Participants	(370)	(370)	(370)

Note. + p < .15, * p < .05, ** p < .01, *** p < .001.

To understand the effect of altruism on PGG contributions, we then analysed two of our baseline specifications, but with DG donations added as a regressor, namely:

$$PGG = k + a * Age + b * Time + c * Time * Age\_Group + d * DG \quad \text{(xvi)}$$

$$PGG = k + a * Age + b * Time + c * Time * Age\_Group + d * DG + e * CE + f * NB \quad \text{(xvii)}$$

When DG donations were added as a regressor to these specifications, donations became the main variable explaining PGG contributions dampening the effects of the other variables, as can be seen in Table E11. But these specifications also highlight a close association between cooperation and altruism, as measured in the PGG and DG.

Table E11

Ordered Probit Estimates of Specifications (xvi) and (xvii)

Variables	Specification (xvi)	Specification (xvii)
Time condition	-1.10^{+}	-0.79
Age	0.02*	0.02^{+}
DG	0.27***	0.26***
Need to Belong	-	0.04
Certainty Equivalent	-	0.19^{+}
Slow*Adolescent	(baseline)	(baseline)
Slow*Early Adult	-0.32^{+}	-0.30
Slow*Adult	-0.40	-0.22
Slow*Older Adult	-1.27*	-1.04^{+}
Fast*Adolescent	0.77	0.47
Fast*Early Adult	0.90^{+}	0.69
Fast*Adult	0.67*	0.46^{+}
Fast*Older Adult	(omitted)	(omitted)
Contribute 0.5	-1.42***	-0.71
Contribute 1.5	-0.30	0.42
Contribute 2.5	0.71**	1.44**
Contribute 3.5	1.70***	2.44***
Participants	(364)	(339)

Note. $^{+}$ p < .15, * p < .05, ** p < .01, *** p < .001.

Basic Data Checks

The following checks display key features of the sample and the data. Table E12 shows how the sample is split across the four age groups used in much of our empirical specifications. The sample

of young adults is slightly larger than the rest, whereas the sample of adults is slightly smaller. The table also highlights that below age 30 the sample included significantly more female participants than male ones. Average age within age groups is also reported in the table. In the young adults group (those aged between 20 and 30) average age was only 22.9 revealing some skewness in the age distribution. Among older adults (those aged between 60 and 80) average age was 65.7 again revealing that our sample had more participants in their sixties than in their seventies.

Table E12

Age Group Frequency and Number of Participants

Age Group	Participants	Frequency	Mean Age	St Dev Age	Female
Adolescent	98	25.1%	15.7	2.1	72
Young Adult	122	31.3%	22.9	2.3	83
Adult	67	17.2%	45.4	9.2	36
Older Adult	95	24.4%	65.7	5.1	47
Age Not Reported	8	2%			

Table E13 establishes that the sample was evenly split between time-pressure and time-delay groups. Further, it documents compliance with the time-pressure treatment by reporting response times quantile by quantile in both the time-pressure and the time-delay conditions. Only 7% of those in the time-pressure group did not comply with the treatment by taking more than 10 seconds to respond. Further, quantile by quantile, those in the time-pressure group took at least 10 seconds less to respond than those in the time-delay group. The slowest responders in the time-pressure group took on average 14 second less to respond than those in the same quantile of the time-delay group. Because of this, participants in the time-pressure group taking more than 10 seconds to respond were retained in the analysis. But results would not be affected by dropping them from the analysis.

Table E13

Number of Participants and Response Times by Quantile in Time-Delay and Time-Pressure Groups

Condition	Participants	1%	5%	10%	25%	50%	75%	90%	95%	99%
Time-Delay	186	11.3	11.6	11.8	12.5	13.7	16.4	21.6	26.9	57.7
Time-Pressure	191	1.2	1.5	1.7	2.1	2.8	4.5	7.9	16.1	43.2
